# Survey dataset on mutualistic interactions among *Euterpe edulis* Mart. (Arecaceae) and floral and frugivorous visitors considering influence of neighborhood plant density and availability of resources

**DOI:** 10.1016/j.dib.2018.11.066

**Published:** 2018-11-16

**Authors:** Jaqueline dos Santos, Isabela Galarda Varassin, Valéria Cunha Muschner

**Affiliations:** a Laboratório de Ecologia Molecular Vegetal, Departamento de Botânica, Setor de Ciências Biológicas, PolitecnicoCentro Politécnico, Universidade Federal do Paraná, Caixa Postal 19031, CEP: 81531-990 Curitiba, PR, Brazil; b Laboratório de Interações e Biologia Reprodutiva, Departamento de Botânica, Setor de Ciências Biológicas, Centro Politécnico, Universidade Federal do Paraná, Caixa Postal 19031, CEP: 81531-990 Curitiba, PR, Brazil; c Programa de Pós-Graduação em Ecologia e Conservação, Setor de Ciências Biológicas, Centro Politécnico, Universidade Federal do Paraná, Caixa Postal 19031, CEP: 81531-990 Curitiba, PR, Brazil

**Keywords:** Palm heart, Pollination, Seed dispersal, Sampling completeness analysis

## Abstract

The data are supporting the research article “Effects of neighborhood on pollination and seed dispersal of a threatened palm” (Santos et al., 2018). We recorded through focal observation mutualistic interactions with floral and frugivorous visitors and 11 individuals of the threatened *Euterpe edulis* palm in Brazil. We thus provide two datasets, one about interactions with floral visitors (Pollination data.xlsx) and other about interactions with frugivorous visitors (Seed dispersal data.xlsx). Both datasets are structured in eight spreadsheets: Two about observed interactions among *Euterpe edulis* palm and floral and frugivorous visitors; two response variables (frequency and assembly composition); four predictor variables (conspecific aggregation, basal area, number of rachillae/fruits, flower in anthesis/ripe fruits). We also report here sampling completeness measured from rarefaction of unique interactions versus interaction events recorded.

**Specifications table**

**Table:** 

Subject area	*Biology*
More specific subject area	*Pollination and seed dispersal ecology*
Type of data	*Excel files with spreadsheets and graphs*
How data was acquired	*Direct observation. The graphs are from R software.*
Data format	*Raw, analyzed.*
Experimental factors	*Data are based on direct observation of floral visitors and frugivorous birds at Euterpe edulis Mart. inflorescences and infructescences, respectively.*
Experimental features	*Data collected were subjected to Distance Matrix Regression Analysis and Principal Coordinate Analysis (PCoA)*
Data source location	*Guaraqueçaba City, Brazil,* S25°10׳56”, W48°17׳55"
Data accessibility	*Data is with this article and Mendeley Data repository*https://doi.org/10.17632/jyttfh4vt4.3
Related research article	*Santos, J., Varassin, I.G., Muschner, V.C. 2018.* Effects of neighborhood on pollination and seed dispersal of a threatened palm. Acta Oecologica 92: 95–101. [1]

**Value of the data**

• Considering *Euterpe edulis* palm is threatened, recorded mutualistic interactions among the palm and its floral and frugivorous visitors can be compared with the records from another area, aiming to conserve and protect *E. edulis* in a broader range.
• The data could compose a major dataset about mutualistic networks.
• Data about mutualistic interactions at the intraspecific level, on small-scale, are important considering the heterogeneity of traits among conspecific and its potential influence on ecological processes.
• Data that enable to evaluate pollination and seed dispersal processes in the same area, in the same population, could help to explain the genetic pattern found for the same population.

## 1 Data

### 1.1 Raw data

We provided data about mutualistic interactions between floral visitors and frugivorous birds and the endangered palm *E. edulis* (11 different individuals for each ecological processes). We collected data through direct observations at Reserva Natural Salto Morato (RNSM), Brazil. It was considered a mutualistic interaction when floral visitors contacted flower reproductive structures and when frugivorous birds ate fruits. Data are presented in two Excel files named “Pollination data.xlsx” and “Seed dispersal data.xlsx” that have eight spreadsheets described below:

#### 1.1.1 Spreadsheet named: 1. interactions

The numbers mean mutualistic interactions recorded through direct observation, among 11 individuals of *E. edulis* and floral visitors (Pollination data.xlsx), and 11 individuals of *E. edulis* and frugivorous visitors (Seed dispersal data.xlsx). *Euterpe edulis* palm trees were named in first column as FIP (Focal Individual sampled for Pollination data.xlsx) or FID (Focal Individual sampled for Seed Dispersal data.xlsx). Each floral and frugivorous visitor was named in first row.

#### 1.1.2 Spreadsheet named: 2. interactions X rachillae / interactions X fruits

Mutualistic interactions recorded (1. interactions, as described above) were multiplied per number of rachillae of inflorescence observed (Pollination data.xlsx) or per number of ripe fruits of infructescence observed (Seed dispersal data.xlsx). The matrix was used to calculate interaction frequency, considering the hours of observation (spreadsheet 3. frequency). *Euterpe edulis* palm trees were named in first column as FIP or FID. Each floral and frugivorous visitor was named in first row.

#### 1.1.3 Spreadsheet named: 3. frequency

The numbers mean interaction frequency divided per observation time for each FIP/FID (last column). The 11 *E. edulis* palm trees were named FIP or FID in first column. Floral and frugivorous visitors were named in first row. Frequency was considered a response variable in the related research article [1].

#### 1.1.4 Spreadsheet named: 4. assembly composition

A binary matrix where 0 (zero) means absence and 1 (one) means presence of floral (Pollination data.xlsx) or frugivorous visitors (Seed dispersal data.xlsx) during observation time for each FIP or FID. *Euterpe edulis* palm trees were named FIP or FID in first column. Floral and frugivorous visitors were named in first row. Assembly composition was considered a response variable in the related research article [1].

#### 1.1.5 Spreadsheet named: 5. conspecific aggregation (CA)

The numbers are average distance (meters) of the three nearest neighbors, meaning a measure for conspecific aggregation, then representing conspecific plant density, for each FIP or FID. *Euterpe edulis* palm trees were named FIP or FID in first column. Conspecific aggregation (CA) was considered a predictor variable in the related research article [1].

#### 1.1.6 Spreadsheet named: 6. basal area (BA)

The numbers are total basal areas (meters) of all woody plants with DBH ≥ 5 cm, within a radius of 5 m from the FIP or FID, then representing heterospecific plant density. *Euterpe edulis* palm trees were named FIP or FID in first column. Basal area (BA) was considered a predictor variable in the related research article [1].

#### 1.1.7 Spreadsheet named: 7. Number of rachillae/fruits (NR/NF)

The numbers mean total of rachillae (NR) per inflorescence (Pollination data.xlsx) observed or total of fruits (NF) per infructescence (Seed dispersal data.xlsx) observed for each FIP/FID. These were used as a measure for resource availability. *Euterpe edulis* palm trees were named FIP or FID in first column. Number of rachillae/fruits (NR/NF) were considered predictor variable in the related research article [1].

#### 1.1.8 Spreadsheet named: 8. flower in anthesis/ripe fruits (FA/RF)

A binary matrix where 0 (zero) means absence and 1 (one) means presence of inflorescences with flowers in anthesis (Pollination data.xlsx) or infructescence (Seed dispersal data.xlsx) with ripe fruits on the three nearest individuals of *E. edulis* from the FIP or FID, respectively. Also, representing a measure for resource availability, there is in 7th column total number of inflorescences with flowers in anthesis (FA; Pollination data.xlsx) or of infructescences with ripe fruits (RF; Seed dispersal data.xlsx) considering the three nearest individuals of *E. edulis* from the FIP or FID, respectively. *Euterpe edulis* palm trees were named FIP or FID in first column. Number of flowers in anthesis/ripe fruits (FA/RF) was considered predictor variable in the related research article [1].

### 1.2 Sampling completeness analysis

**Fig. 1:**
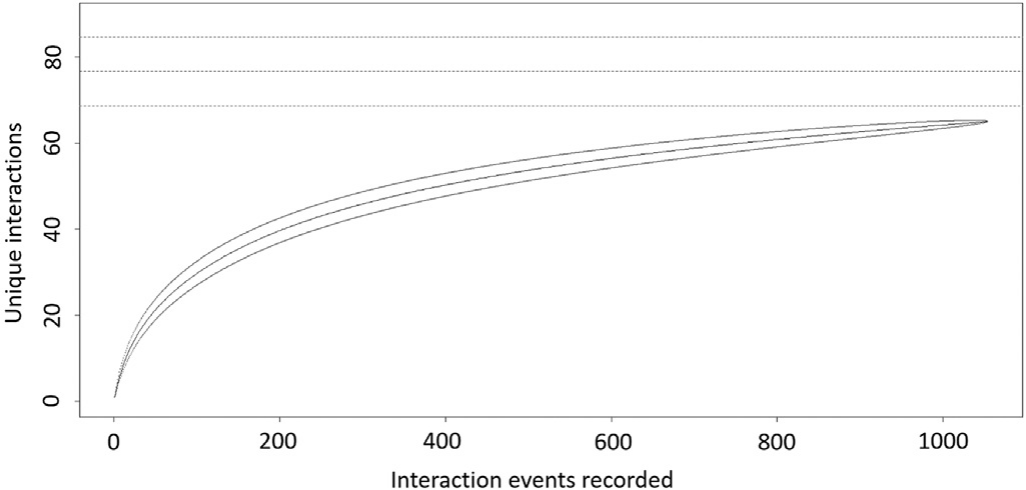
Sampling completeness measured from rarefaction of unique interactions versus interaction events recorded on the focal individuals of *Euterpe edulis* sampled for Pollination data (FIPs) in the Atlantic Forest of Guaraqueçaba, southern Brazil. Horizontal black lines represent the Chao 1 estimate of asymptotic species richness with 95% confidence intervals (dashed lines).

**Fig. 2:**
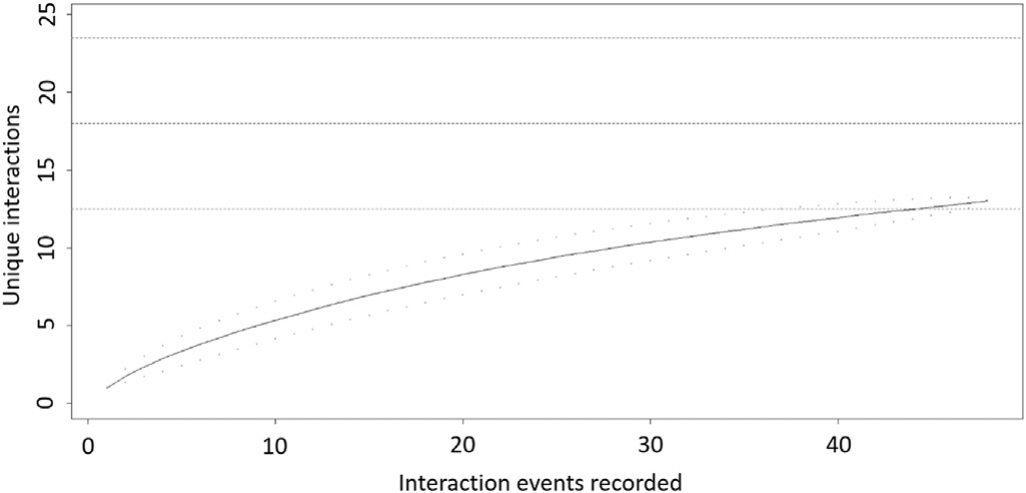
Sampling completeness measured from rarefaction of unique interactions versus interaction events recorded on the focal individuals of *Euterpe edulis* sampled for Seed dispersal data (FIDs) in the Atlantic Forest of Guaraqueçaba, southern Brazil. Horizontal black lines represent the Chao 1 estimate of asymptotic species richness with 95% confidence intervals (dashed lines).

Sampling completeness analysis was measured from rarefaction of mutualistic interactions (spreadsheet named “1. interactions”) among FIP and floral visitors (Fig. 1 and Pollination data.xlsx) and interactions among FID and frugivorous visitors (Fig. 2 and Seed dispersal data.xlsx).

## 2 Experimental design, materials and methods

Experimental design, materials and methods related to raw data and sampling completeness analysis are described at Santos et al. [1].
