# Supplementary material for: Survey dataset on mutualistic interactions among Euterpe edulis Mart. (Arecaceae) and floral and frugivorous visitors considering influence of neighborhood plant density and availability of resources
Source: Data Brief. 2018 Nov 16;21:2015–9. doi: 10.1016/j.dib.2018.11.066 (PMC6258885; doi:10.1016/j.dib.2018.11.066)
Supplement: Supplementary file 1 — Supplementary material [file mmc1.docx]

30 October 2018.

Dear Editor,

This manuscript has not been published and is not under consideration for publication elsewhere. All the authors agree with the submission to *Data in Brief* journal. We have no conflicts of interest to disclose.

Please address all correspondence concerning this manuscript to me at jqln.dos.santos@gmail.com.

Thank you for your time and consideration.

Sincerely,

Jaqueline dos Santos, PhD.

Laboratory of Plant Molecular Ecology, Federal University of Parana, Brazil

Address: Laboratório de Ecologia Molecular Vegetal, Departamento de Botânica, Setor de Ciências Biológicas, Centro Politécnico, Universidade Federal do Paraná, Caixa Postal 19031, CEP: 81531-990, Curitiba, PR, Brazil, E-mail: jqln.dos.santos@gmail.com.
